# Effects of Gestational and Lactational Lead Exposure and High Fat Diet Feeding on Cerebellar Development of Postnatal Rat Offspring

**DOI:** 10.3390/nu15204325

**Published:** 2023-10-10

**Authors:** Jin Seok Seo, Shin Hyo Lee, Hyung-Sun Won, Miyoung Yang, Sang-Seop Nahm, Sung Min Nam

**Affiliations:** 1 Department of Anatomy, College of Veterinary Medicine, Konkuk University, Seoul 05029, Republic of Korea; phoenix_1st@naver.com (J.S.S.); ssnahm@konkuk.ac.kr (S.-S.N.); 2 Department of Anatomy, School of Medicine and Jesaeng-Euise Clinical Anatomy Center, Wonkwang University, Iksan 54538, Republic of Korea; shinhyolee1@wku.ac.kr (S.H.L.); hswon01@wku.ac.kr (H.-S.W.); yangm@wku.ac.kr (M.Y.)

**Keywords:** lead (Pb), high fat diet, cerebellar development, Purkinje cell, rat

## Abstract

Obesity and heavy metals, such as lead (Pb), are detrimental to the adult brain because they impair cognitive function and structural plasticity. However, the effects of co-administration of Pb and a high-fat diet (HFD) on the developing cerebellum is not clearly elucidated. We investigated the effects of Pb exposure (0.3% lead acetate) on developing cerebellum in the pups of an HFD-fed obese rat model. One week before mating, we fed a chow diet (CD) or HFD to the rats for one week and additionally administered Pb to HFD-fed female SD rats. Thereafter, treatment with Pb and a HFD was continued during the gestational and lactational periods. On postnatal day 21, the pups were euthanized to sample the brain tissue and blood for further analysis. Blood Pb levels were significantly higher in HFD-fed rats than in CD-fed rats. Histologically, the prominent degeneration of Purkinje cells was induced by the co-administration of Pb and HFD. The calbindin-28Kd-, GAD67-, NMDAR1-, and PSD95-immunopositive Purkinje cells and inhibitory synapse-forming pinceau structures were significantly decreased following Pb and HFD co-administration. MBP-immunoreactive myelinated axonal fibers were also impaired by HFD but were significantly damaged by the co-administration of HFD and Pb. Oxidative stress-related Nrf2–HO1 signaling was activated by HFD feeding, and Pb exposure further aggravated oxidative stress, as demonstrated by the consumption of endogenous anti-oxidant in HFD–Pb rats. The pro-inflammatory response was also increased by the co-administration of HFD and Pb in the cerebellum of the rat offspring. The present results suggest that HFD and Pb treatment during the gestational and lactational periods are harmful to cerebellar development.

## 1. Introduction

The central nervous system develops from mid-gestation to the postnatal period and then matures into adult brain structures. In rodents, the early postnatal period, the third-trimester equivalent of human pregnancy, is crucial for the developing rodent brain [1]. Developing brain regions, including the cerebellum and hippocampus, are significantly increased by steps of neuroblast generation, migration, neuronal maturation, and synapse formation [1]. As postnatal rodent brains are highly plastic, developing brains in this critical period are highly susceptible to exogenous risk factors: heavy metals, diets, and infectious pathogens. Lead (Pb) is a toxic metal prevalent in the environment through release from natural sources and anthropogenic activities. Another important hazard to the brain is the global pandemic of obesity and subsequent metabolic syndrome [2]. A sedentary lifestyle with easily accessible, high-calorie, sugary, and fatty diets has contributed to the obesity pandemic. Metabolic stress, including hyperglycemia and hyperlipidemia, affects the developing fetal brain in obese mothers, and negative effects persist until adulthood, with a high risk of future development of metabolic syndrome [3,4,5]. Therefore, the HFD-fed obese rodent model is an important tool for risk analysis during the gestational and lactational periods of fetal and postnatal brain development.

Among the various heavy metals, we focused on Pb, which has been widely used in human history for more than 8000 years, such as in adulterating wine, leaded gasoline, lead-based batteries, solder for electronics, leaded paints, cosmetics, ceramics, toys, fishing sinkers, bullets, water pipelines, fertilizers, and electronic waste. It is still a widespread environmental pollutant despite restrictions on its use to reduce its emissions [6,7,8]. According to the ATSDR and the EPA, Pb is the second most dangerous environmental waste worldwide [9]. Compared to the much lower Pb pollution levels in developed countries, Pb pollution and bioaccumulation remain serious problems in highly populated developing countries [10,11,12]. The recent environmental emission of Pb caused a significant health-threatening crisis in the USA, and the US EPA declared ‘war on Pb’ against water pollution with Pb in 2018 [13]. In addition, Pb does not decompose and remains persistent in the environment. Pb can be absorbed by the body via various routes, including inhalation, ingestion, and dermal contact. In humans, the half-life of the absorbed Pb is almost two to three years in the brain and ten to twenty years in the bones. Absorbed Pb can pass through the blood–brain barrier and accumulate in several regions, such as the cerebellum, frontal cortex, and hippocampus [14,15]. A developing immature brain is more vulnerable to Pb-mediated neurotoxicity than a mature adult brain. Children exposed to Pb may develop neurodevelopmental disorders, such as cognitive impairment, autism spectrum disorder, and attention-deficit hyperactivity disorder, with decreased brain volume in the adult stage [10,16,17,18]. Therefore, we selected rat offspring during the fetal and lactation stages to examine the effects of Pb exposure on the developing brain.

Previous investigations have reported that pre- and postnatal exposure to Pb induces cerebellar maldevelopment in litters from Pb-exposed dams [15,19]. To date, the combined effects of HFD feeding and Pb exposure on offspring brain development have not been investigated in these models. We focused on the cerebellum, an important brain area for the coordination of movement, motor learning, control of balance, and equilibrium, because these effects remain to be elucidated. Therefore, we presently investigated whether pre- and postnatal Pb exposure and HFD feeding synergistically act during cerebellar development.

## 2. Materials and Methods

### 2.1. Animals

**Figure 1:**
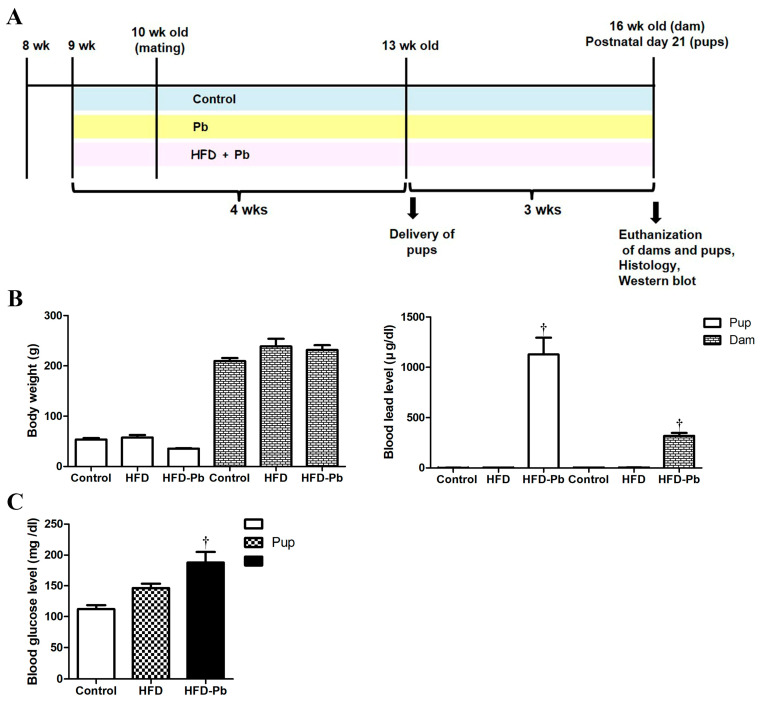
Experimental schedule (**A**) and physiological data (**B**,**C**) of the control, high fat diet (HFD), and HFD plus lead (HFD–Pb) groups. Body weights and blood Pb levels of dams (*n* = 5 per group) and offspring (*n* = 18 per group) on postnatal days (PND) 21 after delivery (**B**). Blood glucose level of offspring on PND21 (**C**) (^†^ *p* < 0.05, control group versus HFD–Pb group). The bars indicate means ± standard errors of mean.

Sprague–Dawley (SD) rats (7 weeks old; 20 females, 7 males) were obtained from Narabiotec (Seoul, Republic of Korea). Animals were housed under identical conditions, i.e., temperature (23 °C), humidity (60%), and 12 h light/dark cycles. After one week of acclimation, rats were used for experiments. They were provided with a chow diet (Purina 5008), or a high-fat diet (HFD, Research Diets D12492; 60% kcal from fat, 5.21 kcal/g), and tap water ad libitum. The experimental design and procedures with animals (Figure 1) were approved by the Konkuk University IACUC (KU20114, approval date 30 July 2020). The researchers carefully conducted all efforts to minimize suffering and stress during the experimental procedures.

### 2.2. Administration of High Fat Diet and Lead Acetate

Adult female rats were separated into three groups: (1) control (*n* = 5 dams), chow diet feeding; (2) HFD (*n* = 5 dams), HFD feeding; and (3) HFD plus Pb (HFD–Pb) (*n* = 10 dams), HFD and Pb co-administration. The experimental design and the exposure regimen of Pb were adopted from previous investigations [15,19]. To induce obesity in female rats, rats allotted to the HFD or HFD–Pb groups were given a high-fat diet. Pb-administered rats received Pb as drinking water; 0.3% lead acetate (Sigma-Aldrich, St. Louis, MO, USA) in distilled water with 0.05% glacial acetic acid (Junsei Chemical Co., Tokyo, Japan). Before mating, female rats in the experimental groups were fed a HFD and Pb for one week. As reported in a previous investigation, pregnancy was confirmed by examining sperm or the vaginal plug [15]. The onset of pregnancy was referred to as Day 0. Then, pregnant females were individually housed for safe delivery and offspring caring. Based on the characteristics of Pb transfer via the placenta and breast milk [20], Pb administration was continued through the gestation and lactation of the offspring until postnatal day (PND) 21 to evaluate the effect of Pb during this critical period. To prevent any influence of litter size and female hormones, we placed five male rat pups per cage (a total of 18 male pups per group), and the remaining offspring were euthanized.

### 2.3. Pb Level Determination in Blood and Measurement of Cerebellar Weight

As described previously [15], atomic absorption spectrometry was performed at Neodin Biovet Laboratory (Seoul, Republic of Korea). Briefly, dams (*n* = 5 per group) and PND21 offspring (*n* = 8 per group) were anesthetized (urethane, intraperitoneal injection of 1.5 g/kg; Sigma-Aldrich) for blood sampling by cardiac puncture. The Pb levels in blood were evaluated by utilizing a PinAcle Perkin-Elmer (Norwalk, CT, USA) Zeeman 5100 atomic absorption spectrometer. For internal and external quality control, Neodin fulfilled the requirement [15]. The spiked Pb sample showed good percentage recovery; detection limits were 0.070 μg/dL, absorption wavelength was 283.3 nm, and r^2^ value of the calibration curve was >0.995. After decapitation, whole brains were rapidly removed and the cerebella were separated, weighed, and kept at −70 °C before analysis.

### 2.4. Histological Analysis and Immunohistochemistry

As described previously [15,19], the remaining PND21 offspring (*n* = 10 per group) were used for histological analysis. Briefly, rats were anesthetized (urethane; Sigma-Aldrich) and transcardially perfused. The cerebella were removed and post-fixed in 4% paraformaldehyde solution (overnight, 4 °C). Sagittally trimmed cerebellar vermis was processed and embedded in paraffin. Histological samples were cut into 5-μm-thick sections using a system for tissue sectioning and staining (HistoCore AUTOCUT and AutoStainer XL, Leica Biosystems, Nussloch, Germany) at the Core Facility for Supporting Analysis and Imaging of Biomedical Materials at Wonkwang University (with support from the National Research Facilities and Equipment Center). Thirty paraffin sections per group (three sections per offspring) were analyzed. Primarily, hematoxylin and eosin (H&E) staining and immunostaining for target proteins were conducted. Briefly, the deparaffinized sections were subjected to antigen retrieval, quenching, blocking, and incubation with primary antibodies (overnight, 4 °C): calbindin-28kD (CB, rabbit, 1:5000; Swant, Bellinzona, Switzerland), glutamic acid decarboxylase 67 (GAD67, mouse, 1:1000; Enzo Life Sciences, Farmingdale, NY, USA), GABA transporter 1 (GABAT1, rabbit, 1:500; Synaptic Systems, Göttingen, Germany), NMDAR1 (rabbit, 1:1000; Millipore, Billerica, MA, USA), myelin basic protein (MBP, rabbit, 1:3000; Millipore), oligodendrocyte transcription factor 2 (Olig2, goat, 1:500; R&D systems, Minneapolis, MN, USA), parvalbumin (PV, rabbit, 1:5000; Swant), postsynaptic density protein-95 (PSD95, rabbit, 1:500; Abcam, Cambridge, UK), or synaptophysin (rabbit, 1:5000; Abcam). Then, the sections were sequentially incubated with biotinylated immunoglobulin G (1:400; Vector, Burlingame, CA, USA) and streptavidin peroxidase complex (1:400; Vector). Finally, the sections were visualized by reaction with 3,3′-diaminobenzidine tetrachloride (Sigma-Aldrich). Some sections were counterstained with hematoxylin. Then, sections were dehydrated and mounted in a toluene-based mounting medium (Thermo Fisher Scientific, Waltham, MA, USA).

Histological analyses were performed by two independent observers blinded to rat treatment under similar conditions. The quantification method employed in this study was conducted as previously described [15,19]. Observations were performed in the anterior (2nd and 5th) and posterior (7th and 8th) lobules of the cerebellar sagittal sections [15].

### 2.5. Western Blotting

As described in previous studies [15,21], immunoblotting for target protein markers was performed using the cerebellar tissue of PND21 pups (*n* = 8 per group). Briefly, to detect the expression level of the protein of interest, frozen cerebellar tissues were homogenized, and the protein concentration was measured by BCA assay (Thermo Pierce^®^ BCA protein assay kit; Thermo Fisher Scientific). Aliquots of rat cerebella (40 μg of protein) were separated by gel electrophoresis and then transferred to polyvinylidene fluoride membranes (Roche, Penzberg, Germany). The membrane was sequentially incubated with a primary antibody (overnight, 4 °C) against either brain-derived neurotrophic factor (BDNF, rabbit, 1:1000; Novus, Littleton, CO, USA), synaptophysin (rabbit, 1:5000; Abcam), nuclear factor erythroid 2-related factor 2 (Nrf2, rabbit, 1:1000; Abcam), superoxide dismutase 1 (SOD1, rabbit, 1:1000; Santa Cruz Biotechnology, Santa Cruz, CA, USA), SOD2 (goat, 1:1000; Santa Cruz Biotechnology), interleukin 1β (IL1β, rabbit, 1:1000; GeneTex, Irvine, CA, USA), tumor necrosis factor α (TNFα, rabbit, 1:2000; Abcam), or heme oxygenase-1 (HO-1, 1:1000; Enzo Life Sciences). Then, the membrane was incubated with a horseradish peroxidase-conjugated secondary antibody (1:2000). Finally, the protein bands were visualized by chemiluminescent reagents (SuperSignal^®^ West Pico Chemiluminescent Substrate, Thermo Fisher Scientific, Rockford, IL, USA). After several rounds of blotting, the blot was densitometrically scanned for quantification and the relative optical density (ROD) of band was analyzed using the NIH ImageJ software (version 1.52a).

### 2.6. Statistical Analysis

The data are shown here as the mean ± standard error of the mean for each group. The statistical differences between these mean values was analyzed using one-way analysis of variance followed by Tukey’s post hoc test using GraphPad Prism 5.01 software (GraphPad Software, Inc., La Jolla, CA, USA). Statistical significance was denoted at *p*-value < 0.05.

## 3. Results

### 3.1. Physiological Parameters and Blood Pb Levels

Primarily, we investigated the effects of HFD feeding and Pb exposure on physiological parameters, such as litter size, sex ratio, body weight, brain weight, cerebellar weight, blood glucose level, and blood Pb level. Body, brain, and cerebellar weights were significantly reduced not by HFD feeding but by HFD and Pb co-exposure in the offspring (Figure 1). We also observed that prenatal and postnatal HFD and Pb co-administration significantly increased blood glucose and Pb levels in offspring, whereas HFD feeding alone did not significantly affect these factors. Additionally, 50% of dams (five out of ten female adult rats) in HFD–Pb group died during perinatal period. In particular, the litter size was significantly reduced by the combined treatment of HFD feeding and Pb treatment, and the sex ratio was greatly imbalanced; as the litter size was thirteen in the control group, eleven in the HFD group, and five in the HFD–Pb group, the sex ratio was near to female offspring 50%/male offspring 50% in the control and HFD groups, and female offspring 63%/male offspring 37% in the HFD–Pb group. The brain (1.3003 ± 0.0155 g) and cerebellar weights (0.161 ± 0.0039 g) were also markedly lower in the HFD–Pb group compared to those in the non-Pb-treated groups: control (brain 1.586 ± 0.0085 g, cerebellum 0.204 ± 0.0024 g) and HFD (brain 1.575 ± 0.0120 g, cerebellum 0.207 ± 0.0036 g).

### 3.2. Effects of HFD Feeding and Pb Exposure on the Developing Cerebellum (H&E Staining and Calbindin-28 Kd Immunohistochemistry)

**Figure 2:**
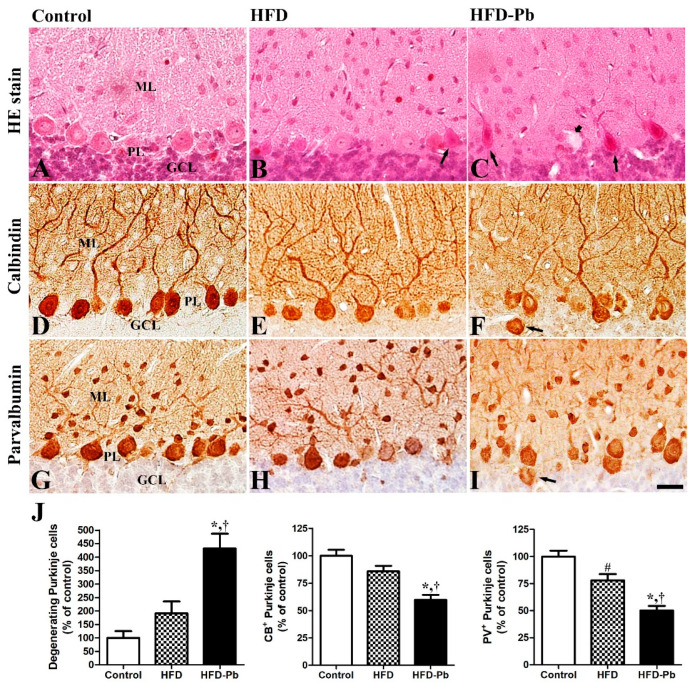
Hematoxylin and eosin staining (**A**–**C**), immunohistochemistry for calbindin (CB) (**D**–**F**), and immunohistochemistry for parvalbumin (PV) (**G**–**I**) in the cerebellum of offspring from control, high fat diet (HFD), and HFD plus lead (HFD–Pb) groups. Note the degenerating Purkinje cells (arrow; **B**,**C**) and vacuole (short arrow), and note that CB was detected in the dendritic branches and somata of Purkinje cells. PV was also detected in some dendrite and cell bodies of Purkinje cells and some cells in the molecular layer of the cerebellar cortex. The numbers of CB- or PV-immunoreactive cells were not significantly changed in the HFD group while they were significantly reduced in the HFD–Pb group. Additionally, ectopic Purkinje cells (arrow; **F**,**I**) were also observed in the granule cell layer of the cerebellar cortex. GCL, granule cell layer; ML, molecular layer; PL, Purkinje cell layer. Bar = 25 μm. (**J**) The numbers of degenerating Purkinje cells, CB-immunoreactive Purkinje cells, and PV-immunoreactive Purkinje cells in the cerebellum are expressed as percentages of the value in the control group (*n* = 10 per group; ^#^ *p* < 0.05, control group versus HFD group, * *p* < 0.05, HFD group versus HFD–Pb group, ^†^ *p* < 0.05, control group versus HFD–Pb group). The bars indicate means ± standard errors of mean.

To identify the combined effects of maternal obesity and Pb exposure on cerebellar development, we conducted H&E staining and immunohistochemistry staining. H&E-stained cerebellar tissues demonstrated that the size was decreased by the combined treatment of HFD and Pb, while macroscopically, the formation of ten lobules and the four main fissures, and microscopically, the lamination of the cerebellar cortex into three layers, namely molecular, Purkinje cell, and granule cell layers, were normally developed in all groups. The number of degenerating pyknotic cells in the Purkinje cell layer was distinctively raised by HFD and Pb co-administration, but not by HFD feeding alone. The Purkinje cell-specific calbindin-28 Kd (CB) immunostaining results demonstrated that HFD feeding did not affect the number of CB-immunopositive Purkinje cells. In the present study, only the coexistence of HFD and Pb exposure during the fetal and postnatal periods markedly impaired the development of CB-immunopositive Purkinje cells by reducing their number. Also, in our preliminary study, the degree of Purkinje cell impairment was prominent in Pb-alone-exposed rats compared to the HFD-fed rats (Figure S1). However, dendritic arborization of the CB-immunopositive Purkinje cells was intensely deteriorated by both a HFD and Pb co-exposure (Figure 2). In addition, developmentally ectopic Purkinje cells were easily observed in the granule cell layer of the offspring from the HFD-fed Pb group.

### 3.3. Effects of HFD Feeding and Pb Exposure on PV Immunoreactivity

We identified PV immunoreaction mainly in the cell bodies and dendrites of Purkinje cells, and in some cells in the molecular and granule cell layers of the cerebellar cortex. HFD treatment alone notably lowered the number of PV-immunopositive Purkinje cells. Compared with the control and HFD-fed groups, the coexistence of HFD feeding and Pb treatment in drinking water further lowered the number of PV-immunopositive Purkinje cells with statistical significance, and their dendritic branches were impaired (Figure 2).

### 3.4. Effect of Pb and Ascorbic Acid on Presynaptic Synaptophysin

**Figure 3:**
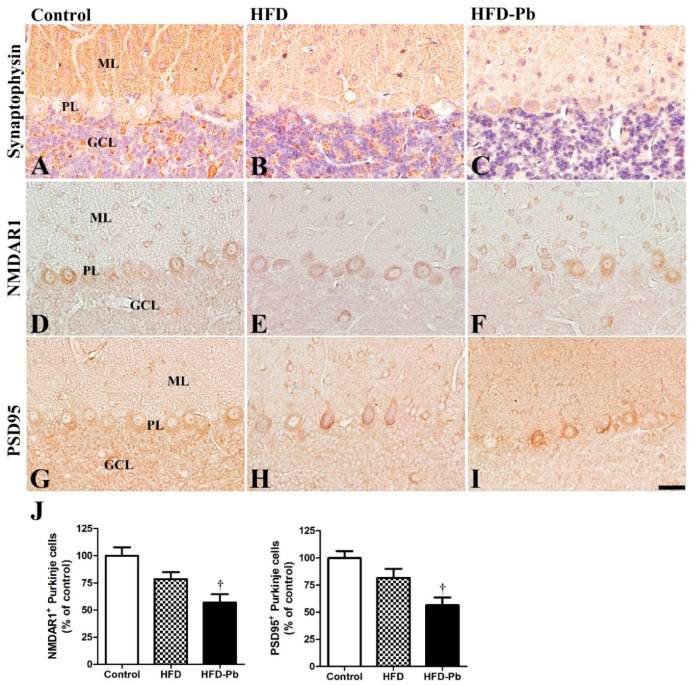
Immunohistochemistry for synaptophysin (**A**–**C**), NMDAR1 (**D**–**F**), and PSD95 (**G**–**I**) in the cerebellum of offspring from control, high fat diet (HFD), and HFD plus lead (HFD–Pb) groups. Note that synaptophysin immunoreactivity was widely detected in the cerebellar cortex, and NMDAR1 and PSD95 were mainly observed in the Purkinje cell layer and in some cells in the molecular and granule cell layers of the cerebellum. GCL, granule cell layer; ML, molecular layer; PL, Purkinje cell layer. Bar = 25 μm. (**J**) The number of NMDAR1-immunoreactive Purkinje cells is expressed as a percentage of the value in the control group (*n* = 10 per group, ^†^ *p* < 0.05, control group versus HFD–Pb group). The bars indicate means ± standard errors of mean.

**Figure 4:**
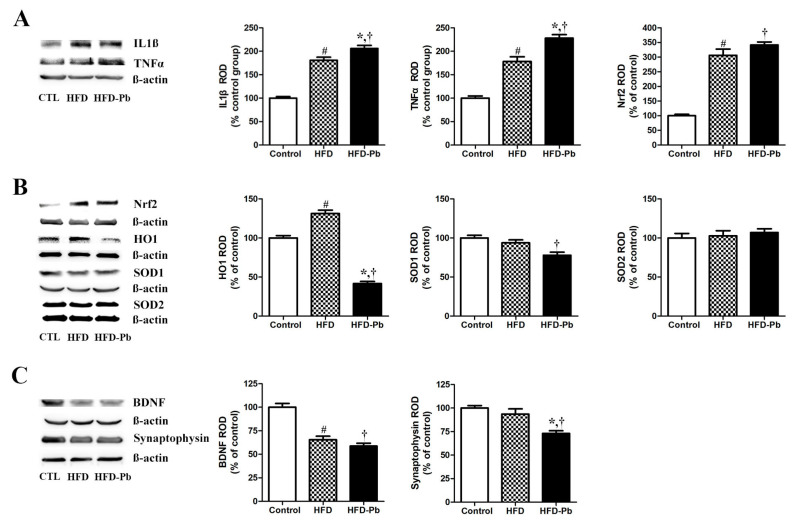
Representative immunoblots and graphs for IL-1β, TNFα, Nrf2, HO1, SOD1, SOD2, synaptophysin, and BDNF (**A**–**C**) in the cerebellum of pups at PND21 from control (CTL), high fat diet (HFD), and HFD plus lead (HFD–Pb) groups. Relative optical density (ROD) of each of these immunoblot bands is expressed as a percentage of the value in the CTL group (*n* = 8 per group; ^#^ *p* < 0.05, control group versus HFD group, * *p* < 0.05, HFD group versus HFD–Pb group, ^†^ *p* < 0.05, control group versus HFD–Pb group). The bars indicate means ± standard errors of mean.

After confirming developmental impairments in Purkinje cell-related markers, we hypothesized that cellular degeneration may be linked to subsequent deficits in synaptogenesis. Here, we further investigated the synaptic marker protein synaptophysin, which is located in presynaptic vesicular membranes and usually demonstrates synaptic formation in the brain [8,10]. We previously observed that synaptophysin was mainly immunostained in the molecular and granule cell layers of the developing cerebellum [8]. The staining and immunoblotting results demonstrated that the expression of synaptophysin in the HFD group was slightly lower than that in the control group, whereas HFD and Pb co-administration further reduced synaptophysin levels in the cerebellum with statistical significance (Figure 3 and Figure 4).

### 3.5. Effect of HFD Feeding and Pb Exposure on NMDAR1 and PSD95

The immunostaining results of postsynaptic NMDAR1 and PSD95 showed similar expression patterns. PSD95 is a scaffolding protein crucial for the gating, trafficking, and cell-surface expression of NMDAR [22]. Subsequently, PSD95 is essential for the maturation of excitatory synapses in developing cerebellum and dendritic spine formation in the cerebral cortex [23,24]. Both NMDAR1- and PSD95-immunoreactivity were mainly observed in the cell body of Purkinje cells, and some were detected in the molecular layer and granule cell layer of the cerebellar cortex. Owing to the combined effect of HFD feeding and Pb exposure, the number of NMDAR1- and PSD95-immunoreactive Purkinje cells was markedly decreased, whereas HFD feeding alone did not affect the number of NMDAR1- or PSD95-immunoreactive Purkinje cells in the cerebellar cortex (Figure 3).

### 3.6. Effect of HFD Feeding and Pb Exposure on γ-Amino-Butyric Acid (GABA)-Synthesizing Enzyme (GAD67) and GABA Transporter 1 (GABAT1)

**Figure 5:**
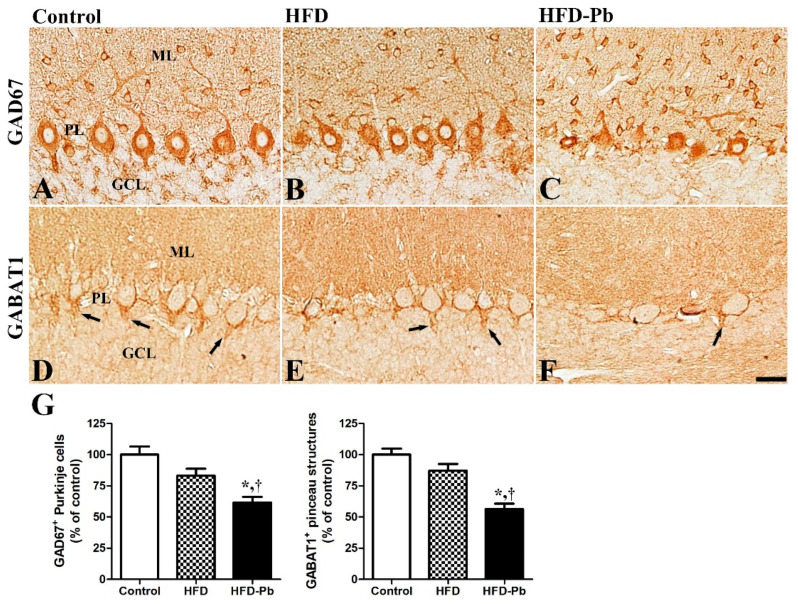
Immunohistochemistry for GAD67 (**A**–**C**) and GABA transporter 1 (GABAT1) (**D**–**F**) in the cerebellum of offspring from control, high fat diet (HFD), and HFD plus lead (HFD–Pb) groups. GAD67-immunopositive Purkinje cells and GABAT1-immunopositive pinceau structures (arrow) were closely associated with the Purkinje cells that were observed in the developing cerebellum. They were not significantly changed in the HFD group but were markedly reduced in the HFD–Pb group. GCL, granule cell layer; ML, molecular layer; PL, Purkinje cell layer. Bar = 25 μm. (**G**) The number of GAD67-immunopositive Purkinje cells and GABAT1- immunopositive pinceau structures is expressed as a percentage of the value in the control group in the developing cerebellum (*n* = 10 per group; * *p* < 0.05, HFD group versus HFD–Pb group, ^†^ *p* < 0.05, control group versus HFD–Pb group). The bars indicate means ± standard errors of mean.

In the cerebellum, the stellate, basket, and Purkinje cells are GABAergic. Stellate and basket cells, classified as interneurons, regulate the activity of Purkinje cells, the only outputs from the cerebellar cortex to the deep cerebellar nucleus and other parts of the brain. In addition, the neurotransmitter GABA is important during brain development because it acts in an excitatory manner to regulate neurogenesis, neuronal proliferation and migration, neuronal differentiation, and neuronal circuit formation in the developing brain [25]. Two important molecules, GAD67 (a GABA-synthesizing enzyme) and GABAT1 (a GABA-uptake transporter), were mainly observed in the Purkinje cell layer in the developing cerebellum. Both GAD67-immunoreactive Purkinje cells and GABAT1-immunoreactive pinceau structures were strongly decreased in the cerebellum of the offspring by the combination of HFD feeding and Pb exposure. GAD67-immunoreactivity was also found in small cells in the molecular layer, where the basket and stellate cells are located. For the structural characteristic, the number of GABAT1-pinceau structures, which enclose the somata and initial axon segment of Purkinje cells, was highly correlated with the number of intact Purkinje cells in the developing cerebellum (Figure 5).

### 3.7. Effect of HFD Feeding and Pb Exposure on MBP and Olig2

**Figure 6:**
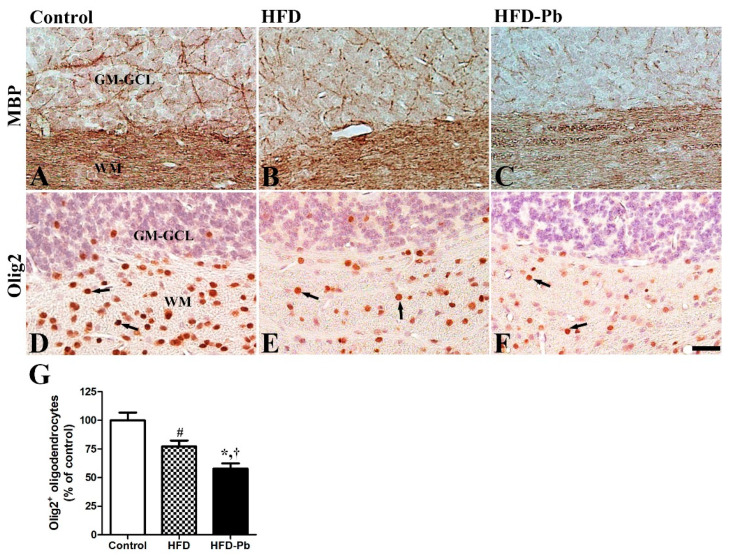
Immunohistochemistry for myelin basic protein (MBP) (**A**–**C**) and oligodendrocyte transcription factor 2 (Olig2) (**D**–**F**) in the cerebellum of offspring from control, high fat diet (HFD), and HFD plus lead (HFD–Pb) groups. MBP-immunopositive myelinated fibers and Olig2- immunopositive oligodendrocytes (arrow) were mainly observed in the cerebellar white matter. The number of Olig2-immunopositive oligodendrocytes was statistically decreased in HFD group and their numbers were further reduced in the HFD–Pb group. GM-GCL, granule cell layer in gray matter; WM, white matter. Bar = 25 μm. (**G**) The numbers of Olig2-immunopositive oligodendrocytes in the cerebellar white matter are expressed as percentages of the value in the control group (*n* = 10 per group; ^#^ *p* < 0.05, control group versus HFD group, * *p* < 0.05, HFD group versus HFD–Pb group, ^†^ *p* < 0.05, control group versus HFD–Pb group). The bars indicate means ± standard errors of mean.

In addition to structural changes in the dendritic branches and somata of Purkinje cells, we investigated the effects of HFD feeding and Pb exposure on the myelinated axonal development and myelin sheath-forming oligodendrocytes in the developing cerebellum. Also, as MBP is the main protein constituent of myelin sheath, we immunostained and alternatively evaluated the morphology of the developing neuronal fibers in the white matter of the cerebellum. MBP-immunoreactive fibers were slightly impaired by HFD feeding, whereas the co-administration of HFD and Pb significantly affected MBP immunoreactivity in the developing cerebellum. Additionally, oligodendrocytes were widely detected in the developing cerebellum, with the majority observed in the white matter. By HFD-feeding, the number of Olig2-immunopositive oligodendrocytes was statistically lowered. The Olig2-immunopositive oligodendrocytes were further reduced in the cerebellar white matter of the HFD and Pb co-administered group, compared to those in the other groups (Figure 6).

### 3.8. Effect of HFD Feeding and Pb Exposure on Nrf2, HO1, SOD1, SOD2, IL-1β, TNFα, and Iba1

We carried out immunoblot analyses to reveal the effect of HFD feeding and Pb exposure on Nrf2 expression (-HO1 signaling) to determine its importance in the protection against oxidative stress. We presently confirmed that HFD feeding increased the expression of Nrf2 and that co-administration of Pb and HFD also increased Nrf2 expression in the whole cerebellum. Similarly, in a recent study, Pb exposure alone mediated oxidative stress by increasing Nrf2 and SOD2 (mitochondrial anti-oxidant) in the developing cerebellum [15]. Histologically, Nrf2 was mainly detected in the intact and degenerating Purkinje cell in the developing cerebellum (Figure S2). The results of Nrf2 immunostaining also supports the HFD-feeding-mediated induction and further increase by HFD–Pb co-administration. Additionally, we found that SOD1 (a cytosolic anti-oxidant) was slightly reduced by HFD feeding, and the Pb–HFD co-treatment further decreased the expression level of SOD1 in the whole cerebellum (Figure 4).

However, the protein expression of HO1 was statistically increased by HFD feeding and was significantly decreased by the co-administration of HFD and Pb. HO1 is regulated by the transcription factor Nrf2 and has anti-inflammatory and anti-oxidative roles. These results suggest that the endogenous anti-oxidant SOD1 and HO1 are decreased in response to the accumulation of oxidative stress by HFD and Pb co-administration.

Additionally, we investigated the expression pattern of the pro-inflammatory cytokines IL-1β and TNFα. We confirmed that both IL-1β and TNFα showed increased patterns in the developing cerebellum with the HFD feeding and Pb exposure, and the degree of increase was significant in HFD–Pb co-administered offspring, compared with HFD-fed offspring (Figure 4). Histologically, Iba1-immunoreactive microglia were detected in all areas of the cerebellum. Iba1 immunoreactivity increased with HFD feeding and was further up-regulated by HFD–Pb co-administration (Figure S2). As reported in other studies, a morphological change in Iba1-immunoreactive microglia was observed; less ramified early postnatal immature microglia increased their arborization by HFD feeding and Pb exposure [26,27]. Present results indicate that HFD feeding and Pb exposure, in dams, induce neuroinflammation in the cerebellum of offspring.

### 3.9. Effect of HFD Feeding and Pb Exposure on BDNF

BDNF is a key neurotrophic factor involved in neuronal development, including growth, differentiation, maturation, survival, and plasticity [28]. BDNF acts as a neurotransmitter modulator and is widely distributed in various brain regions, such as the olfactory bulb, hippocampus, cerebral cortex, and cerebellum [28,29]. In our study, the expression level of BDNF in the cerebellum was significantly reduced by HFD feeding and its level was similarly reduced by the co-administration of HFD and Pb (Figure 4).

## 4. Discussion

As obesity epidemics and blood Pb levels above the Center for Disease Control (CDC) limit are prevalent [2,12,30,31], investigating the combined effects of maternal obesity and Pb exposure on brain development is urgent. In the present study, we focused on the effects of prenatal and postnatal Pb exposure on cerebellar development in the offspring of HFD-fed dams. Blood Pb levels were prominently increased in offspring and dams from the HFD–Pb group, compared with the previous offspring, which were individually treated for Pb [15,19]. Compared with previous studies, HFD and Pb co-administration also significantly reduced physiological parameters, such as body, brain, and cerebellar weights of the offspring [15,19]. The lethality of Pb was significantly increased with HFD and Pb co-administration, compared with that in the Pb-exposed rats; Pb was lethal to 50% of dams in HFD–Pb group and lethal to almost 0% of dams in the Pb group [15]. The developmental neurotoxicity of high blood Pb levels was experimentally confirmed in the present study, and the CDC further lowered the limit of blood Pb levels to less than 5 g/dL in children. This level is harmful to children’s cognitive development; therefore, zero tolerance of blood Pb should be emphasized [31,32]. Interestingly, blood glucose levels were also significantly increased in the HFD–Pb co-administered group compared to those in the HFD-fed and CD-fed groups. In addition to the well-known causes of obesity, such as a sedentary lifestyle and high-calorie food, hyperglycemia in the offspring from the HFD–Pb group can be linked with Pb’s neglected risk of developing metabolic dysfunction by negatively affecting the metabolism of the brain and skeletal system and triggering insulin resistance and obesity [33,34].

H&E staining revealed that HFD feeding during the prenatal and postnatal periods did not significantly affect cerebellar development in the offspring, at least in terms of morphology. However, the co-administration of HFD and Pb significantly caused developmental defects by reducing the number of Purkinje cells in the cerebellum of the offspring. Additionally, the increased number of degenerating Purkinje cells and ectopic Purkinje cells located in the granule cell layer were detected in the HFD–Pb group. A possible explanation of HFD and Pb co-administration-induced structural impairments in the developing cerebellum is hyperglycemia. Previous studies also reported impaired hippocampal neurogenesis and regenerative neurogenesis as subsequent structural impairments from the hyperglycemic or diabetes mellitus model [35,36]. Moreover, the histological analysis of patients with advanced stages of diabetic ketoacidosis corroborates our results by showing a reduction in Purkinje cells in the cerebellum [37]. The results of CB and PV immunohistochemistry were also in line with the H&E staining, and the combined treatment of HFD and Pb was a high-risk factor for the development of the cerebellum compared to the non-teratogenic effect of HFD feeding in the offspring.

During brain development, the sequential expression of GABAergic and glutamatergic receptors is crucial for neural synapse formation [38]. Mature Purkinje cells are the main regulators of output from the cerebellar cortex via the inhibitory neurotransmitter GABA secretion [39]. To elucidate the effects of HFD feeding and Pb exposure on glutamate receptors, GABA-synthesizing enzymes, and GABA transporters in the developing cerebellum, we immunostained the important marker proteins: NMDAR1, GAD67, and GABAT1. In the cerebellar cortex, NMDAR1 and GAD67 were mainly detected in the cell bodies of Purkinje cells; co-administration of HFD and Pb prominently decreased the number of NAMDR1- and GAD67-immunopositive Purkinje cells, whereas HFD feeding itself did not change their numbers. In the present study, PSD95, which regulates the clustering and activity of NMDARs at the glutamatergic excitatory synapses, exhibited a similar pattern of changes in the cerebellum. Similar to the present results, gestational and lactational Pb exposure lowered the number of surviving Purkinje cells in the cerebellum at PND30 [40] and the mRNA expression levels of the GABA receptor subunit and GAD67 in the hippocampus and prefrontal cortex at PND22 [41]. A single platinum injection at PND10 reduced GAD67 levels and impaired Purkinje cell-related synapses in the developing cerebellum [42]. Besides the reduction in the GABA-synthesizing enzyme, the GABAT1-immunoreactive pinceau structure entwining the cell body and axonal initial segments of Purkinje cells was also significantly reduced by HFD and Pb co-administration. Inhibitory basket cells modulate the firing of Purkinje cells by forming pinceau structures with their axonal processes. Compared with the previously reported HFD-induced reduction in GABA in the hippocampus and prefrontal cortex [43], both the synthesis and transport systems of GABA in Purkinje cells were negatively affected by HFD and Pb co-administration, rather than by HFD feeding. In addition to the impairment of glutamatergic and GABAergic receptors in the cerebellum, we focused on another synaptic marker, synaptophysin. Similar to the glutamatergic and GABAergic marker proteins, synaptophysin expression was reduced by HFD feeding and HFD–Pb co-administration. The results presented here indicate that the HFD and Pb co-administration-induced impairment of developing Purkinje cells is linked to deficits in neuronal cell formation and synaptogenesis.

The cerebellum is connected to other brainstem areas and the spinal cord via afferent and efferent nerve fibers because of its critical roles in motor coordination and learning. Among the component fibers, the sole output of the cerebellar cortex is the axons of Purkinje cells which innervate the cerebellar deep nuclei. As co-administration of HFD and Pb reduced the number of Purkinje cells in the cerebellar cortex, we hypothesized that, subsequently to the cell body loss, the myelinated axonal fibers of Purkinje cells also change in the white matter. The combined treatment with HFD and Pb significantly impaired myelinated fibers by reducing MBP immunoreactivity in the cerebellum. Similar to the present results, diabetes also decreased the expression level of MBP in the brain [44]. Myelin sheath-forming oligodendrocytes in the cerebellar white matter were also significantly affected by the co-administration of HFD and Pb. Similarly, we reported the destructive effect of Pb exposure on myelination in the developing cerebellum by reducing MAG and MBP expression [15,19,45]. The reduced number of myelin-forming oligodendrocytes in the cerebellar white matter supports the downregulation of MBP expression following HFD and Pb co-administration. Ma et al. also reinforced our present results by showing the impairment of oligodendrocyte differentiation following Pb intoxication [46].

As HFD feeding and Pb co-exposure negatively affected cerebellar development in offspring, we investigated the mechanism of maldevelopment by focusing on oxidative stress and inflammation. The Nrf2/anti-oxidant response element (ARE) is an important signaling pathway that regulates the expression of oxidative stress-detoxifying genes. We confirmed that HFD feeding increased the expression of the transcription factor Nrf2, and co-administration of HFD and Pb increased its expression in response to accumulating oxidative stress in the developing cerebellum. The downstream target molecule HO1 also showed a similar pattern of change after HFD feeding; however, co-administration of HFD and Pb significantly reduced HO1 expression. As reported in a previous study on pregnant women with diabetes [47], HFD feeding-induced oxidative stress may be protected by HO1 upregulation, and HFD and Pb co-administration significantly consumed HO1, so its expression may be downregulated. A recent study by Liu et al. supported our results by confirming the inhibition of nuclear translocation of Nrf2 and reduced the expression HO1 and NQO1 in the brain [48]. From these results, we suggest that future study investigating the Nrf2 in cytoplasmic and nuclear fraction is warranted. The cytoplasmic anti-oxidant SOD1 is important in neutralizing the oxidative stress generated by HFD and Pb. The overwhelming oxidative stress caused by HFD and Pb co-administration further exhausted endogenous SOD1 in the developing cerebellum. While the expression of anti-oxidants, such as HO1 and SOD1, were affected by HFD feeding and Pb exposure, the expression of SOD2 was not significantly altered by HFD and Pb exposure in the PND21 cerebellum. The present results suggest that the extent of HFD feeding-induced oxidative stress and HFD–Pb co-exposure-induced oxidative stress can be different and that protective responses and mechanisms in cells can be affected differently. Until PND21, the rat offspring obtained their energy resources from their mother’s milk. HFD feeding during the gestational and post-gestational periods affected brain development in the offspring of HFD-fed dams. In contrast to the outstanding negative effects of HFD feeding in adult rodent models, the weak effect in the present rat offspring may be linked to the effects of HFD feeding being indirectly generated and transferred after the completion of hepatic metabolic processes in HFD-fed dams. However, ingested Pb is transferred to the offspring via the placenta, milk, and drinking Pb-containing water.

Along with the oxidative stress-related marker proteins, we confirmed the protein levels of IL-1β and TNFα were prominently up-regulated by HFD feeding. Offspring from dams co-administered with HFD and Pb showed further increases in these pro-inflammatory cytokines in the developing cerebellum. After Iba1 immunostaining, inflammatory cytokines-releasing microglia showed a similar pattern of change with HFD feeding and HFD–Pb co-administration. In line with the present results, White et al. also demonstrated a pro-inflammatory effect of maternal HFD feeding on the prefrontal cortex of offspring [49]. Further studies support the pro-inflammatory effects of Pb exposure in HFD-fed offspring by demonstrating that neuroinflammation is one of the mechanisms of Pb-induced neurotoxicity in the developing cerebellum [15]. As accumulated oxidative stress is a triggering factor of inflammation in the brain [50], HFD and Pb co-administration not only induced severe oxidative stress but also subsequently aggravated neuro-inflammation via further increasing the level of pro-inflammatory cytokines in the cerebellum at PND21.

Additionally, the expression level of BDNF was markedly reduced by HFD feeding, and the co-treatment of HFD and Pb similarly reduced BDNF levels in the developing cerebellum. BDNF is an important regulator of brain developmental processes, including neurogenesis, synaptogenesis, and gliogenesis [28,51]. As Pb is transferred from the dam to the fetus, exogenous BDNF is transferred from the dam to the developing fetal brain in a BDNF knockout mouse model [52]. We suggest that HFD feeding and Pb exposure in dams reduced maternal BDNF, and subsequently, placenta-traversing maternal BDNF also contributed to the reduced level of BDNF in the fetal brain. Both fetal and postnatal BDNF levels may play critical roles in fetal brain development. The decreased expression of BDNF at PND21 may have resulted in the reduction in Purkinje cells and oligodendrocytes in the cerebellum of offspring from the HFD and HFD–Pb groups. As reported previously [53], the reduction in Purkinje cell-related synaptic marker proteins in the present study also reinforces the role of BDNF in synaptogenesis in the developing cerebellum. Similarly, maternal obesity causes a reduction in hippocampal BDNF, resulting in impaired spatial learning in the offspring [54]. In addition, the myelination-promoting effects of BDNF [55] are also linked to the HFD and Pb-mediated reduction in oligodendrocytes and the subsequent impairment in myelination in the developing cerebellum. Based on both the present and previous studies, we suggest that the negative effects of HFD and Pb co-administration are derived from the reduction in BDNF, which is an important regulator of multiple developmental processes of the cerebellum.

As the HFD feeding alone did not cause significant structural impairment in the developing cerebellum, the contribution of Pb to the neuropathology of HFD and Pb co-exposure cannot be neglected. Above all, the degeneration and impairment of Purkinje cells were statistically increased by the HFD–Pb treatment group, compared to the Pb-alone exposure (Figure S1). We speculated that HFD feeding in Pb-exposed rats caused hyperglycemia and exacerbated the neurotoxic effects of HFD and Pb co-administration. In contrast to the HFD feeding alone, blood glucose levels increased significantly with the co-administration of HFD and Pb. Hyperglycemia plays a role in neuronal degeneration [35,36] and acts as a triggering factor by aggravating the vicious cycle of inflammation and oxidative stress [56]. Even though we did not reveal the direct mechanism of accelerated hyperglycemia by HFD and Pb co-exposure, future studies investigating the impacts on the brain–pancreatic axis are warranted to understand the reason for dysregulated blood glucose control [57]. In addition, dietary change or calorie restriction is an effective way of improving glycemic control and body mass index [58,59], which may further mitigate the harmful effects of Pb and HFD co-administration on cerebellar development. Future studies implicating dietary change from HFD to CD during the developmental period are essential to prevent the potential risk of brain maldevelopment.

## 5. Conclusions

Overall, we presently revealed that HFD feeding and Pb co-administration are detrimental to cerebellar development, whereas HFD feeding alone does not cause significant developmental morphological alterations. The co-administration of HFD and Pb remarkably reduced the number of Purkinje cells and impaired subsequent synaptic development by downregulating BDNF and upregulating inflammation and oxidative stress. These results highlight that HFD feeding to mothers who are at a greater likelihood of Pb exposure during gestation and lactation aggravates neurotoxicity and subsequent Pb-mediated cerebellar developmental deterioration in the offspring.

## Data Availability

The data presented in this study are available on request from the corresponding author.

## Supplementary Materials

The following supporting information can be downloaded at: https://www.mdpi.com/article/10.3390/nu15204325/s1, Figure S1. H&E staining of cerebellar tissue from control (A), HFD (B), Pb (C), and HFD–Pb (D) groups. Figure S2. Immunohistochemistry for Nrf2 (A, B, and C), and Iba1 (D, E, and F) in the cerebellum of offspring from control, HFD, and HFD–Pb groups.

## References

[1] Zeiss C.J. (2021). Comparative Milestones in Rodent and Human Postnatal Central Nervous System Development. Toxicol. Pathol..

[2] Grundy S.M. (2008). Metabolic syndrome pandemic. Arterioscler. Thromb. Vasc. Biol..

[3] Glastras S.J., Chen H., Pollock C.A., Saad S. (2018). Maternal obesity increases the risk of metabolic disease and impacts renal health in offspring. Biosci. Rep..

[4] Godfrey K.M., Reynolds R.M., Prescott S.L., Nyirenda M., Jaddoe V.W., Eriksson J.G., Broekman B.F. (2017). Influence of maternal obesity on the long-term health of offspring. Lancet Diabetes Endocrinol..

[5] Page K.A., Luo S., Wang X., Chow T., Alves J., Buchanan T.A., Xiang A.H. (2019). Children exposed to maternal obesity or gestational diabetes mellitus during early fetal development have hypothalamic alterations that predict future weight gain. Diabetes Care.

[6] Levin R., Brown M.J., Kashtock M.E., Jacobs D.E., Whelan E.A., Rodman J., Schock M.R., Padilla A., Sinks T. (2008). Lead exposures in US children, 2008: Implications for prevention. Environ. Health Perspect..

[7] Singh N., Kumar A., Gupta V.K., Sharma B. (2018). Biochemical and molecular bases of lead-induced toxicity in mammalian systems and possible mitigations. Chem. Res. Toxicol..

[8] Zhang B., Huo X., Xu L., Cheng Z., Cong X., Lu X., Xu X. (2017). Elevated lead levels from e-waste exposure are linked to decreased olfactory memory in children. Environ. Pollut..

[9] ATSDR (2019). Substance Priority List. Agency for Toxic Substances and Disease Registry. https://www.atsdr.cdc.gov/spl/index.html.

[10] Hong S.B., Im M.H., Kim J.W., Park E.J., Shin M.S., Kim B.N., Yoo H.J., Cho I.H., Bhang S.Y., Hong Y.C. (2015). Environmental lead exposure and attention deficit/hyperactivity disorder symptom domains in a community sample of South Korean school-age children. Environ. Health Perspect..

[11] Korbecki J., Gutowska I., Chlubek D., Baranowska-Bosiacka I. (2019). Lead (Pb) in the tissues of Anatidae, Ardeidae, Sternidae and Laridae of the Northern Hemisphere: A review of environmental studies. Environ. Sci. Pollut. Res. Int..

[12] Rees N., Fuller R. (2020). The Toxic Truth: Children’s Exposure to Lead Pollution Undermines a Generation of Future Potential.

[13] Roy S., Edwards M.A. (2019). Preventing another lead (Pb) in drinking water crisis: Lessons from the Washington DC and Flint MI contamination events. Curr. Opin. Environ. Sci. Health.

[14] Chibowska K., Korbecki J., Gutowska I., Metryka E., Tarnowski M., Goschorska M., Barczak K., Chlubek D., Baranowska-Bosiacka I. (2020). Pre- and neonatal exposure to lead (Pb) induces neuroinflammation in the forebrain cortex, hippocampus and cerebellum of rat pups. Int. J. Mol. Sci..

[15] Nam S.M., Choi S.H., Cho H.J., Seo J.S., Choi M., Nahm S.S., Chang B.J., Nah S.Y. (2020). Ginseng gintonin attenuates lead-induced rat cerebellar impairments during gestation and lactation. Biomolecules.

[16] Cecil K.M., Brubaker C.J., Adler C.M., Dietrich K.N., Altaye M., Egelhoff J.C., Wessel S., Elangovan I., Hornung R., Jarvis K. (2008). Decreased brain volume in adults with childhood lead exposure. PLoS Med..

[17] Hauptman M., Stierman B., Woolf A.D. (2019). Children with autism spectrum disorder and lead poisoning: Diagnostic challenges and management complexities. Clin. Pediatr..

[18] Lidsky T.I., Schneider J.S. (2003). Lead neurotoxicity in children: Basic mechanisms and clinical correlates. Brain.

[19] Nam S.M., Seo J.S., Go T.H., Nahm S.S., Chang B.J. (2019). Ascorbic acid supplementation prevents the detrimental effects of prenatal and postnatal lead exposure on the Purkinje cell and related proteins in the cerebellum of developing rats. Biol. Trace Elem. Res..

[20] Gulson B., Mizon K., Korsch M., Taylor A. (2015). Revisiting mobilisation of skeletal lead during pregnancy based on monthly sampling and cord/maternal blood lead relationships confirm placental transfer of lead. Arch. Toxicol..

[21] Nam S.M., Seo M., Seo J.S., Rhim H., Nahm S.S., Cho I.H., Chang B.J., Kim H.J., Choi S.H., Nah S.Y. (2019). Ascorbic acid mitigates D-galactose-induced brain aging by increasing hippocampal neurogenesis and improving memory function. Nutrients.

[22] Lin Y., Skeberdis V.A., Francesconi A., Bennett M.V., Zukin R.S. (2004). Postsynaptic density protein-95 regulates NMDA channel gating and surface expression. J. Neurosci..

[23] Losi G., Prybylowski K., Fu Z., Luo J., Wenthold R.J., Vicini S. (2003). PSD-95 regulates NMDA receptors in developing cerebellar granule neurons of the rat. J. Physiol..

[24] Yoshii A., Constantine-Paton M. (2007). BDNF induces transport of PSD95 to dendrites through PI3K-AKT signaling after NMDA receptor activation. Nat. Neurosci..

[25] Wu C., Sun D. (2015). GABA receptors in brain development, function, and injury. Metab. Brain Dis..

[26] Savage J.C., St-Pierre M.K., Carrier M., El Hajj H., Novak S.W., Sanchez M.G., Cicchetti F., Tremblay M.È. (2020). Microglial physiological properties and interactions with synapses are altered at presymptomatic stages in a mouse model of Huntington’s disease pathology. J. Neuroinflamm..

[27] Tress O., Maglione M., May D., Pivneva T., Richter N., Seyfarth J., Binder S., Zlomuzica A., Seifert G., Theis M. (2012). Panglial gap junctional communication is essential for maintenance of myelin in the CNS. J. Neurosci..

[28] Bathina S., Das U.N. (2015). Brain-derived neurotrophic factor and its clinical implications. Arch. Med. Sci..

[29] Nam S.M., Cho I.S., Seo J.S., Go T.H., Kim J.H., Nahm S.S., Chang B.J., Lee J.H. (2019). Ascorbic acid attenuates lead-induced alterations in the synapses in the developing rat cerebellum. Biol. Trace Elem. Res..

[30] Bhurosy T., Jeewon R. (2014). Overweight and obesity epidemic in developing countries: A problem with diet, physical activity, or socioeconomic status?. Sci. World J..

[31] Toscano C.D., Guilarte T.R. (2005). Lead neurotoxicity: From exposure to molecular effects. Brain Res. Brain Res. Rev..

[32] Shefa S.T., Héroux P. (2017). Both physiology and epidemiology support zero tolerable blood lead levels. Toxicol. Lett..

[33] Leff T., Stemmer P., Tyrrell J., Jog R. (2018). Diabetes and exposure to environmental lead (Pb). Toxics.

[34] Rhee S.Y., Hwang Y.C., Woo J.T., Sinn D.H., Chin S.O., Chon S., Kim Y.S. (2013). Blood lead is significantly associated with metabolic syndrome in Korean adults: An analysis based on the Korea National Health and Nutrition Examination Survey (KNHANES), 2008. Cardiovasc. Diabetol..

[35] Dorsemans A.C., Soule S., Weger M., Bourdon E., Lefebvre d’Hellencourt C., Meilhac O., Diotel N. (2017). Impaired constitutive and regenerative neurogenesis in adult hyperglycemic zebrafish. J. Comp. Neurol..

[36] Pintana H., Lietzau G., Augestad I.L., Chiazza F., Nyström T., Patrone C., Darsalia V. (2019). Obesity-induced type 2 diabetes impairs neurological recovery after stroke in correlation with decreased neurogenesis and persistent atrophy of parvalbumin-positive interneurons. Clin. Sci..

[37] Hoffman W.H., Artlett C.M., Zhang W., Kreipke C.W., Passmore G.G., Rafols J.A., Sima A.A.F. (2008). Receptor for advanced glycation end products and neuronal deficit in the fatal brain edema of diabetic ketoacidosis. Brain Res..

[38] Deng L., Yao J., Fang C., Dong N., Luscher B., Chen G. (2007). Sequential postsynaptic maturation governs the temporal order of GABAergic and glutamatergic synaptogenesis in rat embryonic cultures. J. Neurosci..

[39] Teune T.M., van der Burg J., dez Eeuw C.I., Voogd J., Ruigrok T.J. (1998). Single Purkinje cell can innervate multiple classes of projection neurons in the cerebellar nuclei of the rat: A light microscopic and ultrastructural triple-tracer study in the rat. J. Comp. Neurol..

[40] Barkur R.R., Bairy L.K. (2016). Histological study on hippocampus, amygdala and cerebellum following low lead exposure during prenatal and postnatal brain development in rats. Toxicol. Ind. Health.

[41] Neuwirth L.S., Phillips G.R., El Idrissi A. (2018). Perinatal Pb^2+^ exposure alters the expression of genes related to the neurodevelopmental GABA-shift in postnatal rats. J. Biomed. Sci..

[42] Bernocchi G., Bottone M.G., Piccolini V.M., Dal Bo V., Santin G., De Pascali S.A., Migoni D., Fanizzi F.P. (2011). Developing central nervous system and vulnerability to platinum compounds. Chemother. Res. Pract..

[43] Sandoval-Salazar C., Ramírez-Emiliano J., Trejo-Bahena A., Oviedo-Solís C.I., Solís-Ortiz M.S. (2016). A high-fat diet decreases GABA concentration in the frontal cortex and hippocampus of rats. Biol. Res..

[44] Serbedzija P., Madl J.E., Ishii D.N. (2009). Insulin and IGF-I prevent brain atrophy and DNA loss in diabetes. Brain Res..

[45] Nam S.M., Seo J.S., Nahm S.S., Chang B.J. (2019). Effects of ascorbic acid on osteopontin expression and axonal myelination in the developing cerebellum of lead-exposed rat pups. Int. J. Environ. Res. Public. Health.

[46] Ma T., Wu X., Cai Q., Wang Y., Xiao L., Tian Y., Li H. (2015). Lead poisoning disturbs oligodendrocytes differentiation involved in decreased expression of NCX3 inducing intracellular calcium overload. Int. J. Mol. Sci..

[47] Xin G., DU J., Wang Y.T., Liang T.T. (2014). Effect of oxidative stress on heme oxygenase-1 expression in patients with gestational diabetes mellitus. Exp. Ther. Med..

[48] Liu C.M., Tian Z.K., Zhang Y.J., Ming Q.L., Ma J.Q., Ji L.P. (2020). Effects of gastrodin against lead-induced brain injury in mice associated with the Wnt/Nrf2 pathway. Nutrients.

[49] White C.L., Pistell P.J., Purpera M.N., Gupta S., Fernandez-Kim S.O., Hise T.L., Keller J.N., Ingram D.K., Morrison C.D., Bruce-Keller A.J. (2009). Effects of high fat diet on Morris maze performance, oxidative stress, and inflammation in rats: Contributions of maternal diet. Neurobiol. Dis..

[50] Solleiro-Villavicencio H., Rivas-Arancibia S. (2018). Effect of chronic oxidative stress on neuroinflammatory response mediated by CD4^+^T cells in neurodegenerative diseases. Front. Cell Neurosci..

[51] Kowiański P., Lietzau G., Czuba E., Waśkow M., Steliga A., Moryś J. (2018). BDNF: A Key Factor with Multipotent Impact on Brain Signaling and Synaptic Plasticity. Cell. Mol. Neurobiol..

[52] Kodomari I., Wada E., Nakamura S., Wada K. (2009). Maternal supply of BDNF to mouse fetal brain through the placenta. Neurochem. Int..

[53] Langhnoja J., Buch L., Pillai P. (2021). Potential role of NGF, BDNF, and their receptors in oligodendrocytes differentiation from neural stem cell: An in vitro study. Cell Biol. Int..

[54] Tozuka Y., Kumon M., Wada E., Onodera M., Mochizuki H., Wada K. (2010). Maternal obesity impairs hippocampal BDNF production and spatial learning performance in young mouse offspring. Neurochem. Int..

[55] Ramos-Cejudo J., Gutiérrez-Fernández M., Otero-Ortega L., Rodríguez-Frutos B., Fuentes B., Vallejo-Cremades M.T., Hernanz T.N., Cerdán S., Díez-Tejedor E. (2015). Brain-derived neurotrophic factor administration mediated oligodendrocyte differentiation and myelin formation in subcortical ischemic stroke. Stroke.

[56] Vanessa Fiorentino T., Prioletta A., Zuo P., Folli F. (2013). Hyperglycemia-induced oxidative stress and its role in diabetes mellitus related cardiovascular diseases. Curr. Pharm. Des..

[57] Fuente-Martín E., Mellado-Gil J.M., Cobo-Vuilleumier N., Martín-Montalvo A., Romero-Zerbo S.Y., Diaz Contreras I., Hmadcha A., Soria B., Martin Bermudo F., Reyes J.C. (2019). Dissecting the brain/islet axis in metabesity. Genes.

[58] Gannon M.C., Nuttall F.Q. (2006). Control of blood glucose in type 2 diabetes without weight loss by modification of diet composition. Nutr. Metab..

[59] Umphonsathien M., Rattanasian P., Lokattachariya S., Suansawang W., Boonyasuppayakorn K., Khovidhunkit W. (2022). Effects of intermittent very-low calorie diet on glycemic control and cardiovascular risk factors in obese patients with type 2 diabetes mellitus: A randomized controlled trial. J. Diabetes Investig..

