# Supplementary material for: Effects of Gestational and Lactational Lead Exposure and High Fat Diet Feeding on Cerebellar Development of Postnatal Rat Offspring
Source: Nutrients. 2023 Oct 10;15(20):4325. doi: 10.3390/nu15204325 (PMC10609260; doi:10.3390/nu15204325)
Supplement: Supplementary file 1 [file nutrients-15-04325-s001.zip › nutrients-2633596-supplementary.pdf]

Effects of gestational and lactational lead exposure and high fat diet feeding on cerebellar development of post-natal rat offspring

Jin Seok Seo [1], Shin Hyo Lee [2], Hyung-Sun Won [2], Miyoung Yang [2], Sang-Seop Nahm [1] and Sung Min Nam [2,*]

[1] Department of Anatomy, College of Veterinary Medicine, Konkuk University, Seoul 05029, Republic of Korea; phoenix_1st@naver.com (J.S.S.); ssnahm@konkuk.ac.kr (S.-S.N.)

[2] Department of Anatomy, School of Medicine and Jesaeng-Euise Clinical Anatomy Center, Wonkwang University, Iksan 54538, Republic of Korea; shinhyolee1@wku.ac.kr (S.H.L.); hswon01@wku.ac.kr (H.-S.W.); yangm@wku.ac.kr (M.Y.)

* Correspondence: namvet1@wku.ac.kr

Supplementary data

H&E stain

As preliminary study, we conducted histological analysis of the cerebellar tissue of offspring from control, HFD, Pb, HFD + Pb groups (n = 5 pups in each group). Rat pups were anesthetized with urethane (Sigma-Aldrich) and perfused transcardially with 0.1 M phosphate-buffered saline (PBS, pH 7.4) followed by 4% paraformaldehyde in 0.1 M phosphate buffer (pH 7.4). The brains were removed and postfixed in the same fixative for 12 h. Brain tissues were dehydrated with graded concentrations of alcohol and xylene for embedding in paraffin. Five-micrometer-thick sections were serially cut using a microtome (Leica, Wetzlar, Germany) and were mounted on silane-coated slides (Muto-glass, Tokyo, Japan). The cerebellar vermis sections were stained according to routine H&E staining procedure. Data shown here as the means mean ± standard error of the mean for each group. The statistical differences between these mean values were analyzed by two-way analysis of variance followed by Bonferroni's post-tests using GraphPad Prism 5.0 software (GraphPad Software Inc., La Jolla, CA). A P-value < 0.05 was considered statistically significant

Nrf2 and Iba1 immunohistochemistry

Further, we conducted immunostaining to observe the expression of Nrf2 and iba1 in the cerebella of offspring from control, HFD, HFD + Pb groups (n = 5 pups in each group). Deparraffinized brain ections (5-μm-thick) were hydrated with graded concentrations of alcohol and washed with 0.01 M PBS. For antigen retrieval, the sections were placed in 400-mL jars filled with citrate buffer (pH 6.0) and heated in a microwave oven (three heating cycles of 5 min each). Thereafter, the slides were sequentially treated with 0.3% hydrogen peroxide (H_2O_2) in 0.1 M PBS and 10% normal horse serum in 0.1 M PBS. Then, they were incubated with diluted anti-Nrf2 (1:200; Santa Cruz Biotechnology, CA, USA), or Iba1 (1:1,000; Wako, Osaka, Japan) overnight, and subsequently exposed to biotinylated immunoglobulin G (1:400; Vector Labs., Burlingame, CA, USA) and streptavidin peroxidase complex (diluted 1:400, Vector Labs). Then, the sections were visualized by reaction with 3,3′-diaminobenzidine tetrahydrochloride (Sigma, St. Louis, MO, USA). The number of Nrf2-immunoreactive Purkinje cells in the cerebellar cortex was counted using an image analysis system equipped with a computer-based CCD camera (Optimas 6.5 software, CyberMetrics, Scottsdale, AZ, USA). Additionally, the analysis of the Iba1 immunoreactivity in the cerebellum was performed using ImageJ 1.50 software (NIH, Bethesda, MD, USA). The intensity of Iba1 was evaluated by relative optical density (ROD), which was obtained by transforming the mean gray level using the formula: ROD=log (256/mean gray level). Images were calibrated into an array of 512 × 512 pixels and each pixel resolution was 256 gray levels. ROD of the background was determined in unlabeled portions of the sections for correction. A ratio of the ROD was expressed as the percentage of control. Data shown

here as the means mean ± standard error of the mean for each group. The statistical differences between these mean values was analyzed by one-way analysis of variance followed by Bonferroni's post-tests using GraphPad Prism 5.0 software (GraphPad Software Inc., La Jolla, CA). A P-value < 0.05 was considered statistically significant

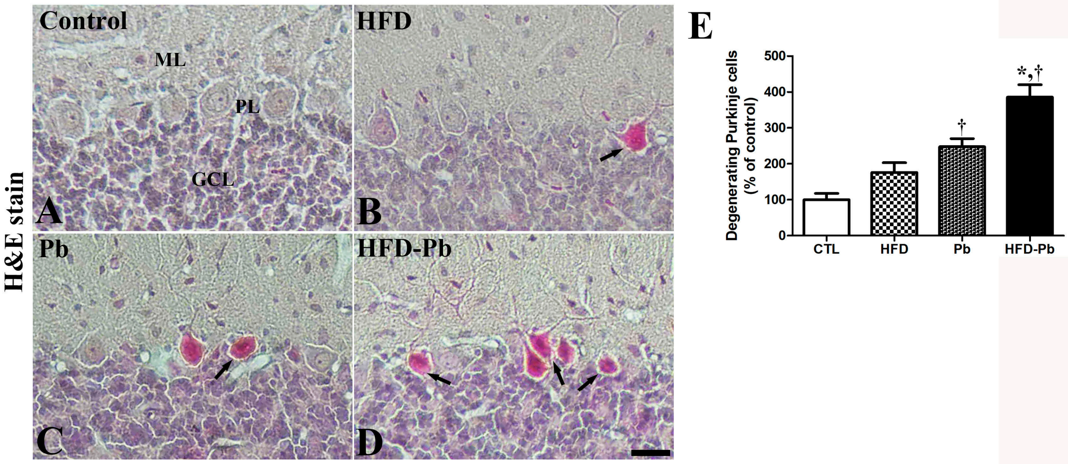

Figure S1. H&E staining of cerebellar tissue from control (A), HFD (B), Pb (C), and HFD-Pb (D) groups. Note that degenerating Purkinje cells (arrow) was detected in the cerebellar cortex. GCL, granule cell layer; ML, molecular layer; PL, Purkinje cell layer. Bar = 25 µm. (E) The numbers of degenerating Purkinje cells in the cerebellum are expressed as percentages of the value in the control group (n = 5 per group; $^{*}P$ < 0.05, CD groups versus HFD groups, $^{\dagger}P$ < *0.05*, non-Pb groups versus Pb groups). The bars indicate means ± standard errors of mean.

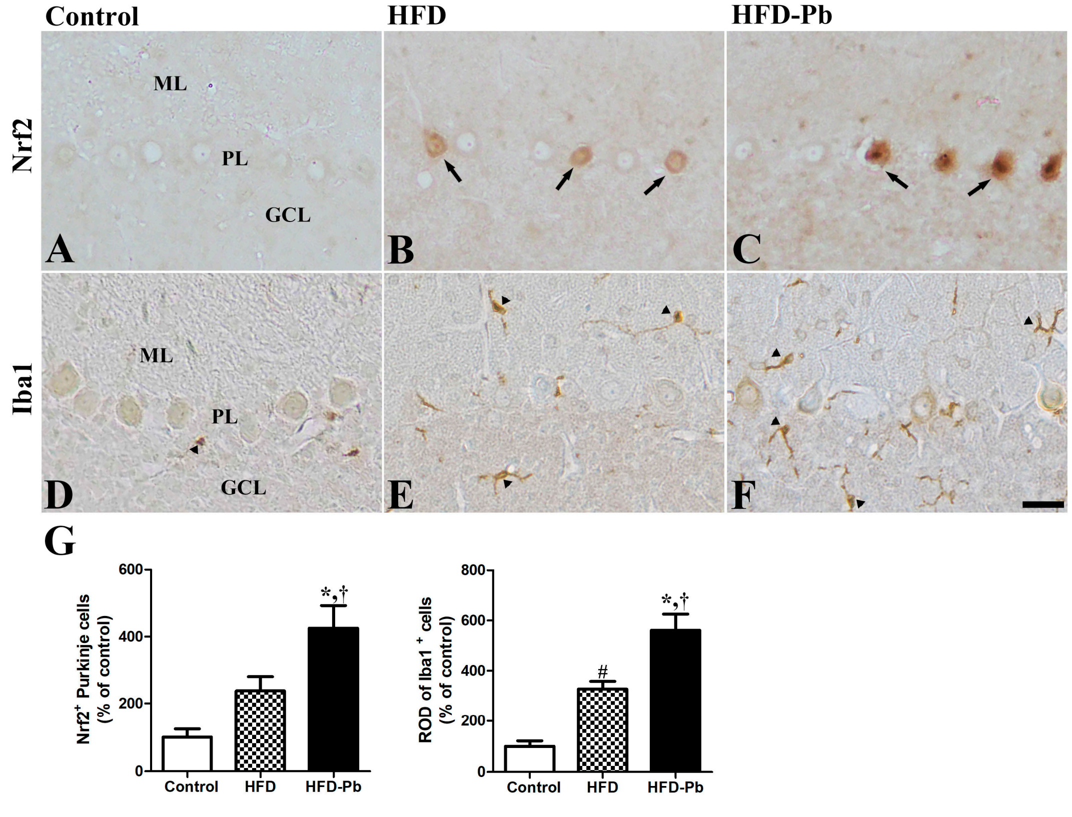

Figure S2. Immunohistochemistry for Nrf2 (A, B, and C), and Iba1 (D, E, and F) in the cerebellum of offspring from control, high fat diet (HFD), and HFD plus lead (HFD-Pb) groups. Note that Nrf2-immunoreactivity was mainly detected in the Purkinje cells (arrow) and Iba1-immunopositive microglia (arrowhead) were widely distributed in all areas of cerebellum. GCL, granule cell layer; ML, molecular layer; PL, Purkinje cell layer. Bar = 25 μm. (G) The number of Nrf2-immunoreactive Purkinje cells and ROD of Iba1-immunoreactiove microglia are expressed as a percentage of the value in the control group (n = 10 per group; $^{\#}P < 0.05$, control group versus HFD group, $^{*}P < 0.05$, HFD group versus HFD-Pb group, $^{\dagger}P < 0.05$, control group versus HFD-Pb group). The bars indicate means ± standard errors of mean.

Raw immunoblot data

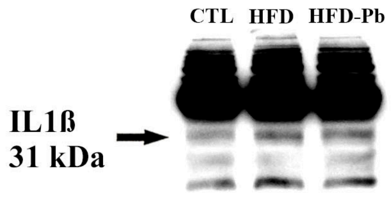

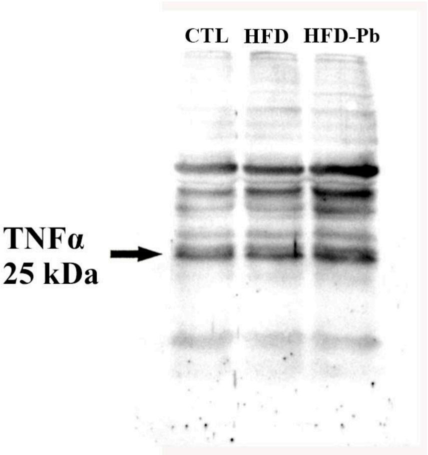

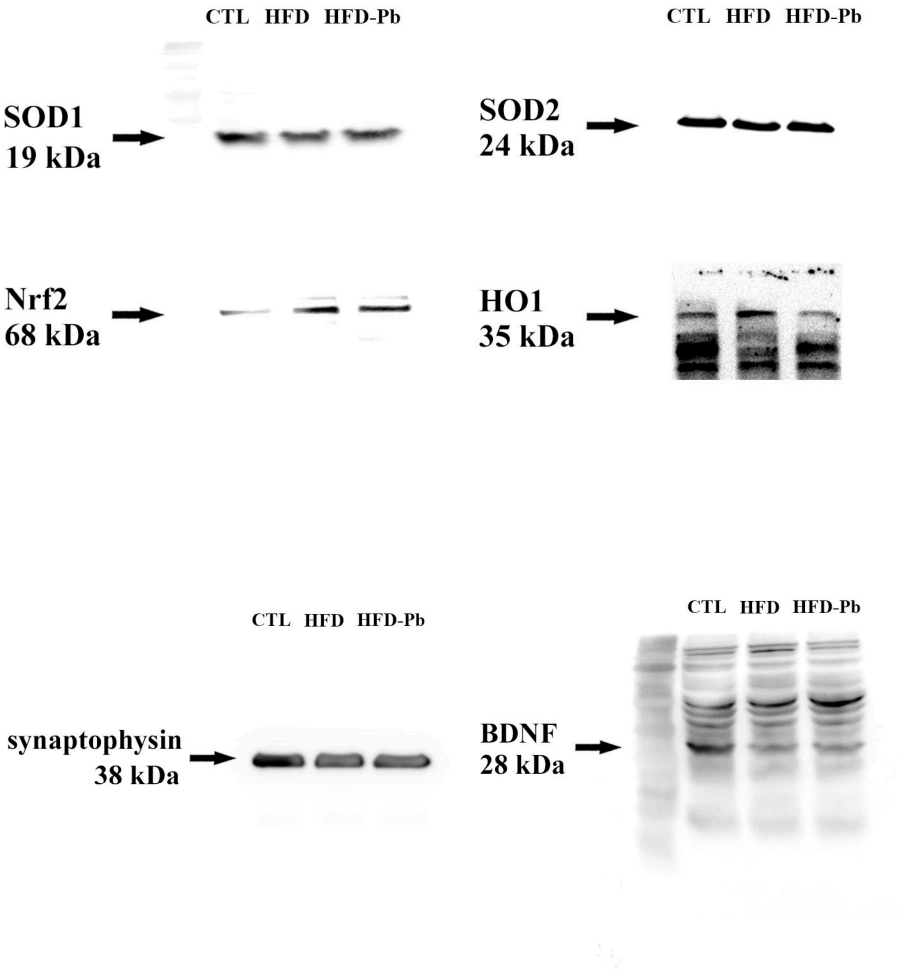

CTL HFD HFD-Pb
SOD1
19 kDa
CTL HFD HFD-Pb
SOD2
24 kDa
Nrf2
68 kDa
HO1
35 kDa
CTL HFD HFD-Pb
synaptophysin
38 kDa
CTL HFD HFD-Pb
BDNF
28 kDa
